# Association analyses of large-scale glycan microarray data reveal novel host-specific substructures in influenza A virus binding glycans

**DOI:** 10.1038/srep15778

**Published:** 2015-10-28

**Authors:** Nan Zhao, Brigitte E. Martin, Chun-Kai Yang, Feng Luo, Xiu-Feng Wan

**Affiliations:** 1 Department of Basic Sciences, College of Veterinary Medicine, Mississippi State University, MS, USA; 2 Institute for Genomics, Biocomputing & Biotechnology, Mississippi State University, MS, USA; 3 School of Computing, Clemson University, Clemson, SC, USA

## Abstract

Influenza A viruses can infect a wide variety of animal species and, occasionally, humans. Infection occurs through the binding formed by viral surface glycoprotein hemagglutinin and certain types of glycan receptors on host cell membranes. Studies have shown that the α2,3-linked sialic acid motif (SA2,3Gal) in avian, equine, and canine species; the α2,6-linked sialic acid motif (SA2,6Gal) in humans; and SA2,3Gal and SA2,6Gal in swine are responsible for the corresponding host tropisms. However, more detailed and refined substructures that determine host tropisms are still not clear. Thus, in this study, we applied association mining on a set of glycan microarray data for 211 influenza viruses from five host groups: humans, swine, canine, migratory waterfowl, and terrestrial birds. The results suggest that besides Neu5Acα2–6Galβ, human-origin viruses could bind glycans with Neu5Acα2–8Neu5Acα2–8Neu5Ac and Neu5Gcα2–6Galβ1–4GlcNAc substructures; *Galβ* and *GlcNAcβ* terminal substructures, without sialic acid branches, were associated with the binding of human-, swine-, and avian-origin viruses; sulfated *Neu5Acα2*–*3* substructures were associated with the binding of human- and swine-origin viruses. Finally, through three-dimensional structure characterization, we revealed that the role of glycan chain shapes is more important than that of torsion angles or of overall structural similarities in virus host tropisms.

Influenza A viruses infect a wide range of hosts, such as humans, sea mammals, swine, bats, and avian, equine, and canine species^1^,^2^,^3^,^4^. The carbohydrates or glycans in host cells serve as the receptors for influenza viruses and are key to successful virus entry, the first step in influenza infection^5^. The structures of these glycan receptors have been shown to be unique in animal hosts and even within different tissues in the same host, and these unique glycan structures determine host and tissue tropisms of influenza A viruses.

In humans, glycans with α2,6-linked sialic acid (SA2,6Gal) are detected more plentifully in the upper respiratory tract than the lower respiratory tract. SA2,6Gal and SA2,3Gal (α2,3-linked sialic acid) are heterogeneously distributed in the human nasopharynx and bronchi. The expression of SA2,3Gal is greater than that of SA2,6Gal in the respiratory tract of young children^6^,^7^. In avian species, SA2,3Gal and SA2,6Gal are distributed within respiratory and intestinal tracts. Although SA2,3Gal are mostly found in waterfowl, it is possible that SA2,3Gal and SA2,6Gal could be expressed very differently in terrestrial birds^8^,^9^,^10^,^11^. Swine express both SA2,3Gal and SA2,6Gal in the respiratory tract, but SA2,6Gal are abundant in the upper trachea and bronchi, and SA2,3Gal are more abundant in the lower respiratory tract^10^,^12^,^13^,^14^. The presence of both SA2,3Gal and SA2,6Gal (e.g. Neu5Acα2–3Gal and Neu5Acα2–6Gal) in swine could allow these animals to be susceptible to avian-origin and human-origin influenza A viruses; thus swine have been proposed as “mixing vessels” for influenza viruses^12^.

By presenting multiple glycans or glycoconjugates printed on a single slide, the glycan microarray technique has offered high-throughput analyses of the glycan-binding profile of influenza viruses^15^,^16^. Glycan microarray has become a routine experimental tool for characterizing the receptor-binding profiles of influenza viruses^17^. To date, >500 influenza virus–related glycan microarray data entries have been deposited in the Consortium for Functional Glycomics (CFG) glycan microarray database^18^, and this number is still increasing. Glycan microarray profiling of influenza virus pandemic strains has shed light on the receptor-binding specificities of their hemagglutinin (HA). For example, such analyses revealed that the 2009 influenza A(H1N1)pdm09 pandemic virus bound to α2,6-linked and to a large range of α2,3-linked sialyl sequences^19^,^20^,^21^,^22^. Moreover, glycan microarray analysis has been widely used to study receptor recognition and host tropism of influenza virus mutants^23^,^24^,^25^,^26^,^27^,^28^. In addition to providing data on Neu5Acα2–3Gal and Neu5Acα2–6Gal, glycan microarray analysis also provided data on other complicated glycan substructures. In one study, structural topology (i.e., two glycan chain shapes, one cone-like and the other umbrella-like) was reported to be related to SA2,3Gal and SA2,6Gal during influenza virus–receptor interactions^29^. On the other hand, during a glycan microarray screening, influenza A viruses were shown to bind receptors other than SA2,3Gal and SA2,6Gal, although such bindings have not been confirmed by interventional experiments^22^,^30^. For example, influenza A (H1N1) virus can bind α2,8-linked polysialyl sequences^22^. Nevertheless, it is still unclear what specific substructures or moieties in host receptors determine influenza virus host tropisms.

To better understand structural specificities for glycan binding, Cholleti *et al.*^31^ developed an algorithm called GlycanMotifMiner, or GLYMMR, that is frequently used with subtree mining to identify motifs for protein–glycan interactions for a given glycan microarray data entry. Porter *et al.*^32^ applied a clustering algorithm to identify glycan substructures with high intensities in the glycan array data. More recently, we developed a novel quantitative structure–activity relationship (QSAR) method to analyze glycan array data; the method focuses on glycan substructure features by applying PLS regression and selection functions to the glycan microarray data^33^. Another frequent glycan structure mining of influenza virus data also detected sulfated glycan motifs increased viral infection^34^. However, none of the above methods were designed for large-scale glycan microarray data analysis that integrates multiple microarray data entries for a particular research interest. Particularly, statistic-based motif identification methods rely on pre-defined hypothesis and could not discover unexpected and infrequent ones. Feature selection strategies for the regressions of glycan microarray data have not considered modeling multiple microarrays. Thus, a computational method is needed to characterize glycan substructure motifs by utilizing the information across multiple datasets, especially glycan microarray data across various platforms, and this method must be able to tolerate the noises within and across glycan microarray data.

The relationship between host receptors (glycan substructures) and influenza A viruses (e.g., viruses with different host origins) can be naturally formulated as a computational problem of data integration plus association rule mining. Therefore, in this study, we firstly applied a PLA regression on individual glycan microarray data entries as normalization and then used association rule analysis on extracted glycan substructure features to identify motifs for influenza virus host tropisms. In addition to SA2,3Gal and SA2,6Gal, results showed that glycan substructures with SA2,8SA, non-sialic acid saccharides (Galβ and GlcNAcβ terminal substructures), and sulfated SA2,3Gal could contribute to influenza host tropism differently. Additional computational modeling demonstrated that, for trisaccharide substructures, a shape angle formed by mass centers of three residues, instead of linkage torsion angles, may determine the overall glycan chain shapes and thus distinguish glycans with SA2,3Gal from those with SA2,6Gal or SA2,8SA. These findings may imply a more general property caused by glycan terminals than just by sialic acid with different linkages during influenza – glycan binding.

## Methods

**Figure 1:**
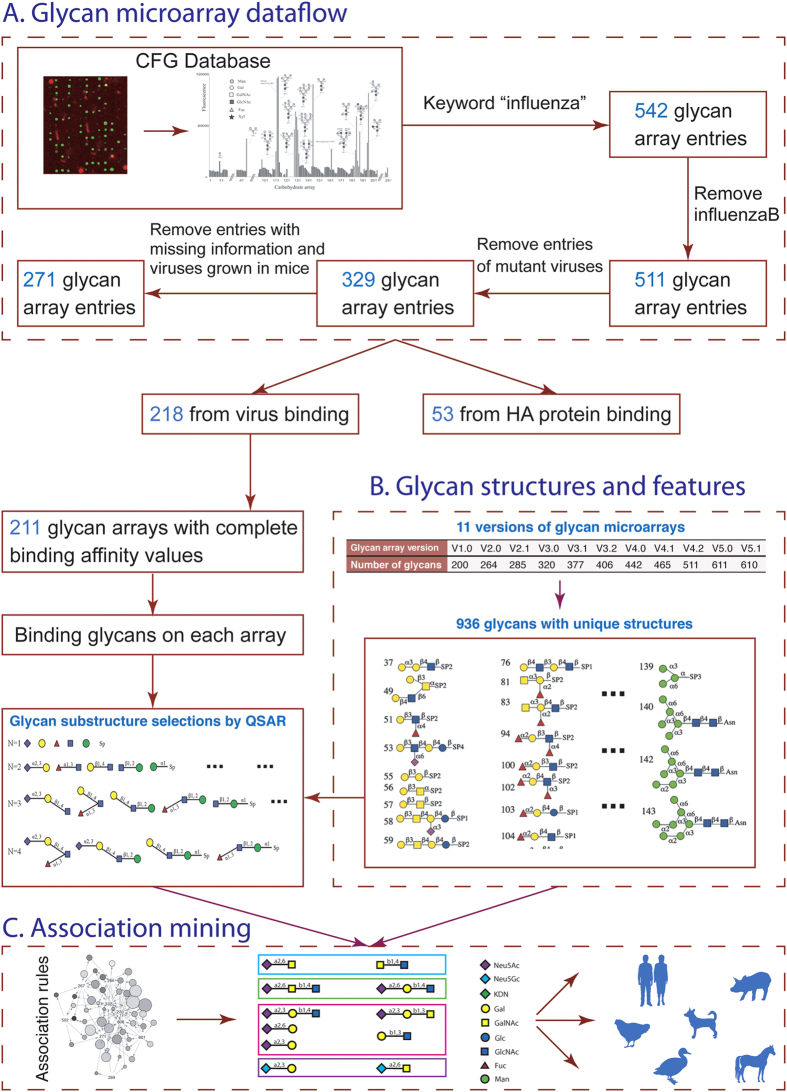
Flowchart of the computational analysis protocol. (**A**) Glycan microarray data collection for influenza virus bindings. (**B**) Glycan structure organizations and substructures’ feature extraction and selection. (**C**) Association rule mining on viral host labeled substructure feature vectors. CFG, Consortium for Functional Glycomics; HA, hemagglutinin; QSAR, quantitative structure–activity relationship.

Figure 1 shows a simplified flowchart of the computational strategy we used, with glycan microarray data, to identify host-specific glycan substructures. In brief, we collected and integrated glycan microarray datasets (Fig. 1A), defined and extracted substructures from glycans (Fig. 1B), and applied association rule mining to identify the influenza viruses’ specific glycans and their substructures (Fig. 1C).

### Datasets

#### Collection of influenza A virus–specific glycan microarray data

A computational script was written to automatically retrieve glycan microarray datasets from CFG^18^ by using the keyword “influenza.” A total of 542 glycan microarray entries were retrieved, of which 324 were excluded from the final dataset: 31 entries for influenza B viruses, 182 for mutant viruses, 51 for mouse-adapted strains, 53 for HA recombinant proteins, and 7 for microarrays with incomplete binding affinity values. The remaining 218 entries were for influenza A virus–specific glycan microarray datasets with complete binding affinity values. These datasets, which were used for further analyses (Table 1), consisted of influenza A viruses of human origin (n = 154), waterfowl origin (n = 17), terrestrial bird origin (n = 13), canine origin (n = 6), and swine origin (n = 21). The metadata associated with these datasets, including CFG entry identification codes, investigators’ names, influenza virus sample names, glycan array version, raw array binding files, and host species, are listed in Table S1 in the supporting information.

#### Integration of glycan microarray datasets

In the CFG database, the datasets were generated by using 11 versions of glycan microarrays, each of which had a different number of glycan entries. For example, version 1 had 200 glycans, whereas version 5.1 had 611 glycans. However, most glycans present in earlier glycan microarray version are present in later versions. To facilitate the data analyses across different datasets, we merged all microarray versions into one with 936 unique glycans (Table S2) and generated a single matrix for the 211 data entries (211 viruses × 936 glycans, Table S3). Glycan-binding affinities (i.e., fluorescent signal values) in our dataset were assigned to corresponding elements in the matrix. The elements for which there was no corresponding affinity value among the 936 glycans were assigned a “not available” value and excluded from the glycan substructure feature selection.

### Glycan substructure feature extraction

Glycan substructures were defined as described elsewhere^33^. Specifically, mono-, di-, tri-, and tetrasaccharide substructures were extracted from 936 glycans as features. These extractions resulted in 249 monosaccharide, 738 disaccharide, 1,198 trisaccharide, and 1,477 tetrasaccharide substructures (Tables S4–S7). The fluorescent signal value for the corresponding glycan on the array was assigned as the binding affinity for each individual substructure. Only fluorescent signal values ≥2,000 were considered as effective numbers in regression, and those <2,000 were treated as background noise. Next, a partial least squares (PLS) regression and feature selection algorithm (QSAR^33^) were adapted to select the features predominating glycan binding from an influenza virus–specific glycan microarray dataset (see details in Supplementary Information). This PLS regression was performed four times for each single data entry from our 211 glycan microarrays by using four sets of substructure feature definitions (mono-, di-, tri-, and tetrasaccharides). Each feature vector was finally labeled according to the host origin of the influenza A viruses used in the glycan microarray experiments (i.e., human, swine, canine, waterfowl, or terrestrial bird [chicken, quail, and turkey] host).

### Association rule mining for selected glycan substructures

We formulated the detection of host-specific glycan substructures as an association-mining task (see more details in Supplementary Information), where we let items 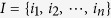 represent a set of items and let 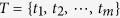 be a set of transactions forming a database. An association rule, 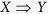, where 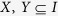, is usually interpreted to mean that when the items in 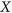 exist, those in 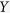 also occur at a certain confidence level^35^. Here, for our glycan microarray dataset, transactions *T* were the data derived from influenza virus–specific glycan microarray entries, so *m* = 211; the substructure features *X* derived from glycans on the array by previous PLS-β selection and the labeled features *Y* with host origin will form *I*. Given a rule 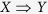, the *confidence* is defined as 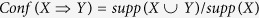, where *sup* (*X*) is the *support* of item set 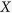. The *support* was defined as the proportion of transactions in the dataset, which contains the item set. Another measurement, *lift*, is the ratio of the observed *support* and was defined as 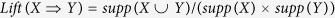
35. Therefore, we expected to obtain interesting association rules with high confidences (≥80%), high lifts (has a lift value ≥1^36^), and low supports (≥0.005, infrequent but potentially interesting) to supply highly probable, unexpected, and infrequent conclusions. We adapted the *Apriori* algorithm implemented in R^37^ to infer these host substructure–specific associations. Moreover, during the mining process, redundant rules were also removed by defining super rules as redundancy. A super rule is a rule with the same or lower lift value, where the left hand side, *X*, contains more items than a previous rule, but still results in the same right hand side, *Y*. Last, we kept only satisfied rules, which were filtered by leaving only those with terminal saccharides on the substructure features.

### Three-dimensional structural modeling and analysis

#### Structural characterization for terminal glycan saccharides

To understand the structural determinants for a specific glycan associated with certain influenza A virus, we compared the spatial relationship between six terminal trisaccharide features derived from data mining. These six features were (Neu5Acα2–6)-(6Galβ1–4)-4GlcNAc (PDB^38^ accession number: 3UBN^39^), (Neu5Acα2–8)–(8Neu5Acα2–8)–8Neu5Ac (3HMY^40^), (Neu5Acα2–3)–(3Galβ1–4)-4GlcNAc (3UBQ^39^), (Neu5Gcα2–3)–(3Galβ1–4)–4GlcNAc (4POT^41^), (Galβ1–4)–(4GlcNAcβ1–3)–3Gal (2XRS^42^), and (Galβ1–4)(Fucβ1–3)–(3,4GlcNAcβ1–3)–3Gal (1SL5^43^). The following three geometric measurements were calculated:

- (1) *The angle formed by the mass centers of three saccharides*. We calculated the angle formed by the mass centers of three saccharides as a measurement of the glycan chain’s turning shape.
- (2) *The root-mean-square deviation (RMSD).* Given two glycan substructures, each containing the terminal saccharide, we superimposed the corresponding atoms on the six-membered rings of the terminal saccharides. From there, while keeping the terminal saccharides superimposed, the following two values of RMSD were measured: RMSD2 and RMSD3. Using the standard formula of calculating RMSD from two sets of six-membered ring atoms 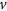 and 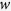: 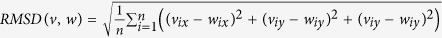, where *n* = 6, RMSD2 was calculated for the two saccharides linked directly to their respective terminal saccharides. If both glycan substructures had a third saccharide, RMSD3 was then calculated for the third pair of saccharides.
- (3) *ϕ and ψ torsion angles*. We calculated the *ϕ* and *ψ* torsion angles for each linkage between two adjacent residues. Glycosidic torsions were defined by only heavy linkage atoms as 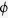 = O5–C1–O_n_–C_n_ and *ψ* = C1–O_n_–C_n_–C_n-1_^44^. Accordingly, a trisaccharide substructure has two linkages with two sets of torsions.

#### Three-dimensional structures for protein–glycan interactions

To demonstrate structural interactions between influenza viruses and glycan substructures at the molecular level, we used four HA protein crystal structures. These structures were from viruses with different host origins (A/California/04/09 H1N1, human origin, SA2,6Gal specific; A/swine/Iowa/15/1930 H1N1, swine origin, SA2,6Gal specific; A/Canine/Colorado/06 H3N8, canine origin, SA2,3Gal specific; A/Vietnam/1203/2004 H5N1, avian origin, SA2,3Gal specific) and were obtained from PDB^38^ data entries, 3LZG^45^, 1RVT^46^, 4UO5^47^, and 2FK0^20^, respectively. We docked these HA proteins with corresponding glycan substructures (6SLN [α2–6-sialyl-*N*-acetyllactosamine], analogous to Neu5Acα2–6Galβ1–4GlcNAc, and 3SLN [α2–3-sialyl-*N*-acetyllactosamine], analogous to Neu5Acα2–3Galβ1–4GlcNAc) and highlighted conserved and diverse regions on the receptor-binding pocket (amino acid residues 98, 133–138, 153, 183, 188–195, 221–228 by H3 numbering). Substructures, (Neu5Acα2–8)–(8Neu5Acα2–8)–8Neu5Ac and (Galβ1–4)–(4GlcNAcβ1–3)–3Gal, were also simulated against bound 6SLN and 3SLN to be docked at the receptor-binding pocket of the human-origin and the avian-origin viral HAs respectively. The HA-glycan docking was conducted by following three steps: 1) an initial structure was obtained by superposing the structures of HA and a glycan analog against a native HA 3D structure with glycan; 2) an energy minimization with 500 steps of conjugation and 500 additional steps of steepest descent was performed by using the AMBER force field^48^ at a GROMACS^49^ dynamic simulation process; and 3) the final complex structure was obtained after an binding free energy repairing by using the FoldX software^50^ at simulation temperature of 298K and without hydrogen atoms.

## Results

### Influenza virus–specific features derived from glycan microarray data by PLS regression and feature selection

Certain saccharide residues are enriched at glycan substructures contributing to influenza virus binding. In the integrated dataset of glycan microarrays with 936 unique glycans, the glycans with influenza virus–binding affinities mostly consist of sialic acids (Neu5Ac and Neu5Gc), galactose (Gal), N-acetylgalactosamine (GalNAc), glucose (Glc), N-acetylglucosamine (GlcNAc), fucose (Fuc), and mannose (Man). Table 2 summarizes the number and percentage of glycan substructures that have these saccharides by each one of the four substructure feature definitions on the glycan microarrays (i.e., mono-, di-, tri-, and tetrasaccharides) and thus reflects their existence according to the microarray design. Table 3 lists the same distribution values of influenza virus–specific substructures selected by PLS regression and illustrates that only a small portion of glycan substructures (73/249 monosaccharides, 230/738 disaccharides, 322/1,198 trisaccharides, and 320/1,477 tetrasaccharides) was determined to contribute to a binding signal of ≥2,000 with influenza viruses. All PLS-selected substructure features are also summarized in Table S8.

A comparison of data in Table 2 with that in Table 3 shows that Neu5Ac, Neu5Gc, Gal, and GlcNAc were more abundant in the glycan substructures contributing to influenza virus binding. For example, Neu5Ac appeared in 9.59% of the monosaccharides, 11.3% of the disaccharides, 18.0% of the trisaccharides, and 31.9% of the tetrasaccharides when QSAR was used to select significant glycan substructures for influenza virus binding (Table 3), compared with 4.82%, 6.10%, 7.68%, and 10.9%, respectively, of all the glycan substructures from microarrays (Table 2). Similar differences were observed for Neu5Gc, Gal, and GlcNAc. These findings suggest that influenza virus–specific glycan substructures are prone to have these four saccharides. Nevertheless, when QSAR was used, glycan substructures with GalNAc, Glc, Fuc, and Man were equally or less frequently correlated with influenza virus binding than those on the glycan arrays (Tables 2 and 3), which indicates a limited contribution to influenza binding by substructures with these saccharides.

### Host-specific glycan substructures derived from association rule mining

**Figure 2:**
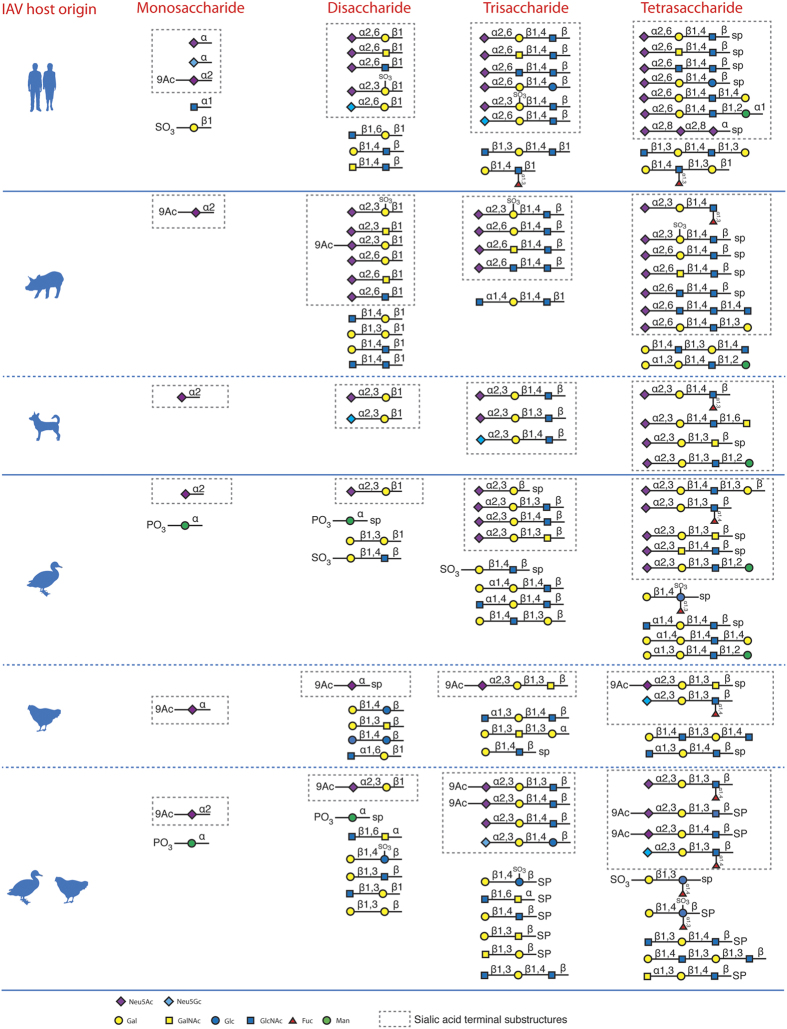
Host-specific glycan substructures detected by association rule mining. Human-associated, mammal (swine and canine)-associated, and avian (waterfowl and terrestrial bird)-associated terminal substructures.

To understand the specific substructures associated with each influenza A virus, we performed associate rule analyses across 211 influenza virus–specific glycan microarray data. On the basis of their host origins, the 211 influenza A viruses were categorized into human (n = 154), canine (n = 6), swine (n = 21), waterfowl (n = 17), terrestrial (i.e., chicken, quail, and turkeys, n=13), and avian (waterfowl plus terrestrial birds, n = 30). The association analysis results (summarized in Fig. 2 and Table S9) illustrate the specific substructures being associated with each of six host origins; these associations aid in our understanding of the key substructures that determine influenza host and tissue tropisms.

#### Neu5Acα2–8Neu5Acα2–8Neu5Ac and Neu5Gcα2–6Galβ1–4GlcNAc substructures contribute to the glycan binding with human-origin influenza A viruses

**Figure 3:**
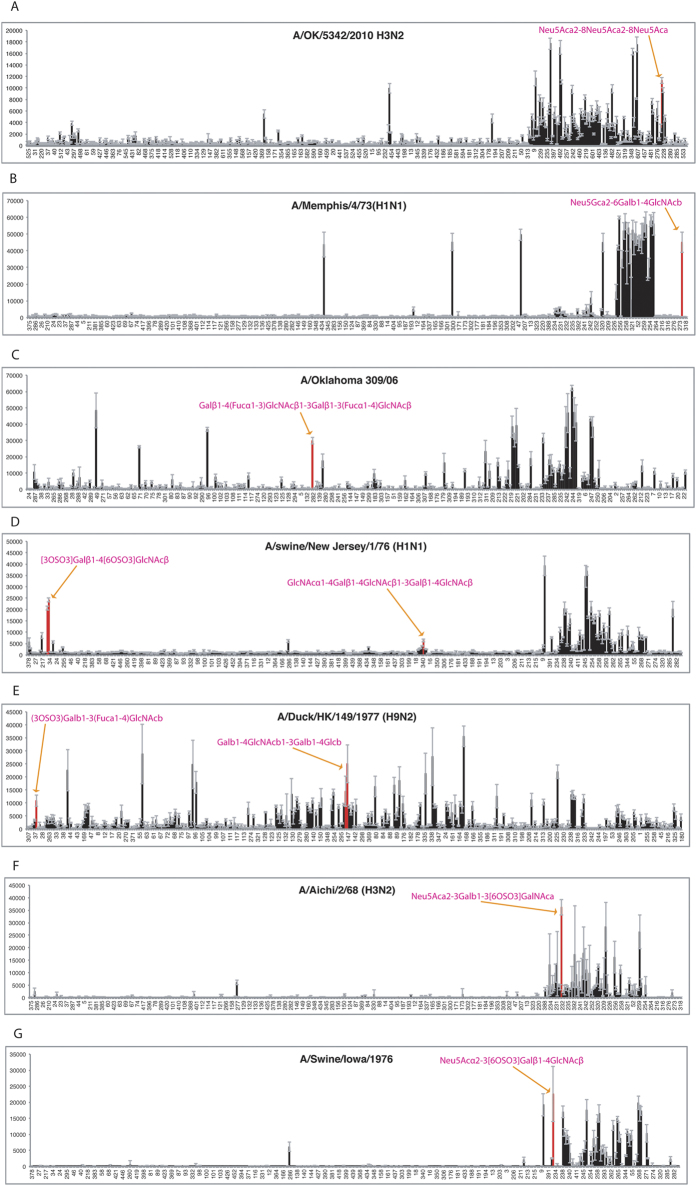
Case studies: individual glycan microarrays of influenza A viruses with interesting binding motifs. (**A**) Human-origin virus A/OK/5342/2010 binds glycan Neu5Acα2–8Neu5Acα2–8Neu5Ac. (**B**) Human-origin virus A/Memphis/4/73 binds glycan Neu5Gcα2–6Galβ1–4GlcNAc. (**C**) Human-origin virus A/Oklahoma 309/06 shows binding ability to glycan with Galβ1–4(Fucα1–3)GlcNAcβ1–3Gal substructure. (**D**) Swine-origin virus A/swine/New Jersey/1/76 shows binding ability to glycans with GlcNAcα1–4Galβ1–4GlcNAc substructure. (**E**) Waterfowl-origin virus A/Duck/HK/149/1977 shows binding ability to glycans with Galβ1–4GlcNAcβ1–3Gal substructure. (**F**) Human-origin virus A/Aichi/2/68 binds to sulfated glycan with α2,3-linked sialic acid terminals. (**G**) Swine-origin virus A/Swine/Iowa/1876 binds to sulfated glycan with α2,3-linked sialic acid terminals.

In addition to the reported α2,6-linked sialic acid glycan substructures (with a Neu5Acα2–6 terminal), which were detected multiple times (28 rules in Table S9) to be associated with human-origin influenza A viruses (Fig. 2), Neu5Acα2–8Neu5Acα2–8Neu5Ac (frequency = 0.0299, confidence = 1.00, lift = 1.44) and Neu5Gcα2–6Galβ1–4GlcNAc (frequency = 0.0479, confidence = 1.00, lift = 1.44) substructures were also found to be associated with human-origin influenza A viruses’ glycan binding (Fig. 2 and Table S9). In Fig. 3A,B, as case studies, two glycan microarray data entries of human-origin viruses demonstrate the significantly high binding affinities to these substructures separately. Specifically, virus A/OK/5342/2010 binds to glycan Neu5Acα2–8Neu5Acα2–8Neu5Ac and A/Memphis/4/73 to glycan Neu5Gcα2–6Galβ1–4GlcNAc. Although Neu5Gc has not been reported to be present in human respiratory track tissues, human-origin viruses may have the binding ability to α2,6-linked Neu5Gc substructures on glycan microarrays. This observation also suggests that, in addition to Neu5Acα2–6 terminal, other sialic acids with either α2–6 or α2–8 linkages may be recognized by human-origin influenza A viruses.

#### Galβ and GlcNAcβ terminal substructures, in addition to Sialic Acids, associated with the glycan binding of human-, swine-, and avian-origin influenza A viruses

The α2,3-linked and α2,6-linked sialic acid glycan substructures were identified as predominated glycan binding motifs of all types of influenza A viruses (Fig. 2). However, it was interesting to observe that glycan substructures with Galβ and GlcNAcβ terminals were detected to be associated with human-, swine-, and avian-origin viruses. These two terminal saccharides are usually followed by β1,3-, β1,4-linked, and occasionally α1,3-, α1,3-linked Gal, GlcNAc, or GalNAc (e.g., Galβ1–4(Fucα1–3)GlcNAcβ1–3Gal with human-origin virus binding: frequency = 0.0359, confidence = 0.857, lift = 1.23; GlcNAcα1–4Galβ1–4GlcNAc with swine-origin virus binding: frequency = 0.012, confidence = 1.00, lift = 9.28; Galβ1–4GlcNAcβ1–3Gal with avian-origin virus binding: frequency = 0.0419, confidence = 1.00, lift = 5.96) (Fig. 2 and Table S9). Moreover, these Galβ and GlcNAcβ terminal substructures could result in influenza viruses binding independently, since many glycans with either Galβ or GlcNAcβ terminals, out of the 936 unique glycans on microarrays, do not contain any sialic acid saccharides (Table S2). And there are individual microarray data entries showing influenza viruses bind to Galβ or GlcNAcβ terminal glycans with no other branches at the same time. For instance, in Fig. 3C,E, human-origin virus A/Oklahoma 309/06, swine-origin virus A/Swine/New Jersey/1/76, and waterfowl-origin virus A/Duck/HK/149/1977 all have relatively high binding signals on their individual glycan microarrays. We then conclude that, without existing sialic acid saccharide residues, glycans having Galβ or GlcNAcβ terminal could serve as potential receptors for influenza A virus.

#### Sulfation causes Neu5Acα2–3 substructures associated with the glycan binding of human- and swine-origin influenza A viruses

As with human-origin influenza A viruses, not surprisingly, multiple substructures (19 rules in Table S9) with Neu5Acα2–6 were identified as being associated with the glycan binding of swine-origin influenza A viruses. In addition, the substructures with Neu5Acα2–3 were also associated with swine-origin influenza A viruses (Fig. 2). However, interestingly, the substructures with Neu5Acα2–3 terminals are usually sulfated on the following saccharide residues, when they were identified to be associated with either human- or swine-origin influenza A viruses. For example, Neu5Acα2–3(6OSO3)Galβ1–4GlcNAc was associated with human-origin (frequency = 0.0719, confidence = 0.80, lift = 1.51) and swine-origin (frequency = 0.0179, confidence = 1.00, lift = 9.28) viruses separately (Fig. 2 and Table S9). As shown in Fig. 3F,G, a human-origin virus A/Aichi/2/68 and a swine-origin A/Swine/Iowa/1976 both have their highest binding affinity to sulfated glycans with α2,3-linked sialic acid terminals (Neu5Acα2–3Galβ1–4(6OSO3)GlcNAc, and Neu5Acα2–3(6OSO3)Galβ1–4GlcNAc). This association rule linking sulfated Neu5Acα2–3 glycans and human-, swine-origin virus binding may support a unique role of sulfation during human and swine adaptation of avian-origin influenza A viruses.

### Consensus among the influenza virus–specific glycan substructures

**Figure 4:**
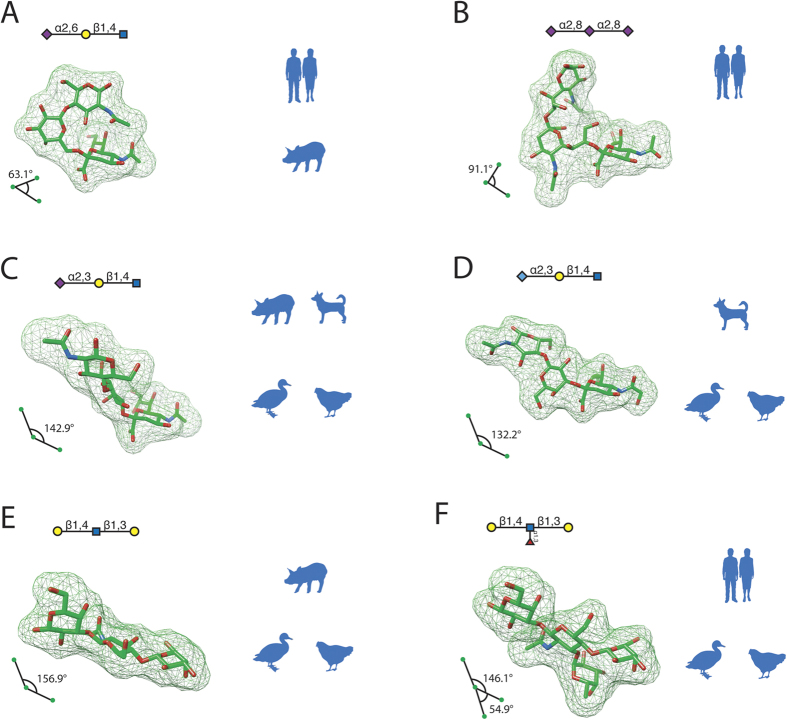
Three-dimensional structures of host-specific glycan substructures. (**A**–**D**) Representative structures of host-specific trisaccharide substructures with sialic acid terminals. The shape angles were calculated by the mass centers of three residues. (**E–F**) Structures of trisaccharide substructures with Gal (non-sialic acid) terminal saccharide.

**Figure 5:**
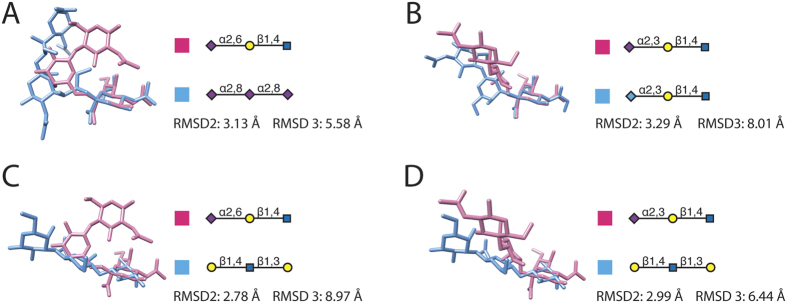
Superposition of terminal saccharide residues and the root-mean-square deviations (RMSDs) between the second (RMSD2) and the third (RMSD3) residues.

To identify common features from the substructures associated with different hosts, we compared the structural similarity among them by calculating, as described in Methods, the angle formed by three mass centers of all residues for trisaccharide substructures, RMSD2 and RMSD3, and ϕ and ψ torsion angles of linkages for the six representative glycan substructures (Fig. 4). In addition, superposition images of these glycan substructures are shown in Fig. 5.

#### 3D structural characterization for glycan substructures with sialic acid terminals

As shown in Fig. 4A,D, we obtained four trisaccharide three-dimensional structures, of which one has SA2,6Gal terminal, one has SA2,8SA terminal, and two have SA2,3Gal terminals. Three observations were made from the substructures. First, the residue mass centers for the SA2,6Gal substructure formed acute angles (63.1°), for the SA2,8SA substructure formed an angle of 91.1°, and the mass centers for both α2,3-linked substructures formed obtuse angles (142.9° and 132.2°). This observation suggests that SA2,6Gal and SA2,3Gal substructures are fundamentally different from each other on saccharide chain shapes and thus could lead to virus host tropism, in which human influenza viruses recognize glycans with SA2,6Gal terminals, canine and avian viruses recognize glycans with SA2,3Gal terminals specifically, but swine viruses can recognize and bind to both shapes. Moreover, the α2,8-linked polysialyl substructure with a right angle shares a more similar turning shape to the one of SA2,6Gal and then may cause the human-origin influenza virus binding.

Second, the all-against-all RMSD values for these glycan substructures indicate that none of the substructures with sialic acid terminals are similar on the basis of both RMSD2 and RMSD3 values, if we define similar saccharide structures by using RMSD2 smaller than 3 Å and RMSD3 smaller than 5 Å (Table 4, Fig. 5). This finding shows that shape angles formed by residue mass centers are not the sole factor for glycan structural diversity.

The third observation involves the linkage torsion angles of these four representative host-specific trisaccharides (Table 5). It is shown that, although most torsions of linkage 2 share similar values and hence do not contribute much to virus host types, both ϕ and ψ angles of linkage 1 distribute variously and indicate the shape-forming roles of α2,6, α2,8 and α2,3 linkages with terminal sialic acids. In particular, the linkage 1 ϕ angle values of these four trisaccharides, combined with their shape angles formed by residue mass centers, could shed some light on the relationship between glycan geometric shapes and influenza virus host types. On one hand, when trisaccharides with SA2,6Gal or SA2,8SA have angles of acute shapes (Fig. 4A,B), a positive ϕ angle (e.g., 71.32° of Neu5Acα2–6Gal or 55.05° of Neu5Acα2–8Neu5Ac in Table 5) might be necessary to make the glycan associated with human host type; however, the association might not be unique because the positive ϕ angle might also result in an association with swine viruses. On the other hand, when trisaccharides with SA2,3Gal have angles of obtuse shapes (Fig. 4C,D), different terminal residues (i.e., Neu5Ac and Neu5Gc) form ϕ angles with different values (e.g., a −59.47° of Neu5Acα2–3Gal and a 50.95° of Neu5Gcα2–3Gal in Table 5). This observation illustrates that an obtuse angle of α2,3-linked trisaccharides is sufficient, but not necessary, for a glycan to associate with non-human-origin viruses and that a positive ϕ torsion angle at linkage 1 may make the trisaccharides associated with canine- and avian-origin viruses only. Furthermore, all four trisaccharides, except Neu5Acα2–8Neu5Acα2–8Neu5Ac, have linkage 1 ψ angles of similar negative values and therefore do not show a clear relationship with virus host types.

In summary, the structural characteristics of glycan trisaccharides with sialic acid terminals might be associated with influenza virus host tropisms. For example, it seems that the shape angle formed by residue mass centers plus the linkage 1 ϕ torsion angle, not just torsion angles themselves, might suggest certain glycan structural patterns associated with influenza virus host tropism.

#### 3D structural characterization for glycan substructures with a Gal terminal

For glycan trisaccharide substructures with a Gal, we found two additional representative three-dimensional structures of glycans that had the same terminal residues and linkages associated with viruses of different host-origins: Galβ1–4GlcNAcβ1–3Gal and the fucosylated one Galβ1–4(Fucα1–3)GlcNAcβ1–3Gal (Fig. 4E,F). The Galβ1–4GlcNAcβ1–3Gal backbone of both substructures formed similar obtuse angles (156.9° and 146.1°), while the additional fucose residue formed a 54.9° angle with the Galβ1–4GlcNAcβ1 terminal. This additional turning angle introduced by fucosylation may lead to the human-origin virus binding (Fig. 4F). Next, we measured RMSDs between the Galβ1–4GlcNAcβ1–3Gal substructure and the ones with sialic acid terminals (Table 4 and Fig. 5C,D). Although Galβ1–4GlcNAcβ1–3Gal has similar RMSD2 values with Neu5Acα2–6Galβ1–4GlcNAc (2.78 Å) and with Neu5Acα2–3Galβ1–4GlcNAc (2.99 Å), a smaller RMSD3 value (6,44 Å) with Neu5Acα2–3Galβ1–4GlcNAc showed its better structural similarity to the SA2,3Gal motif. Thus, this finding might suggest that the glycan substructure Galβ1–4GlcNAcβ1–3Gal does not have to maintain a sialic acid terminal to share a structural similarity with the avian–virus-binding motif (SA2,3Gal). Last, nevertheless, torsion angles of both linkages of Galβ1–4GlcNAcβ1–3Gal have unique values comparing to those of sialic acid substructures and therefore do not support any relationships with various virus bindings.

### Structural conservation of receptor binding pocket in influenza A viruses

**Figure 6:**
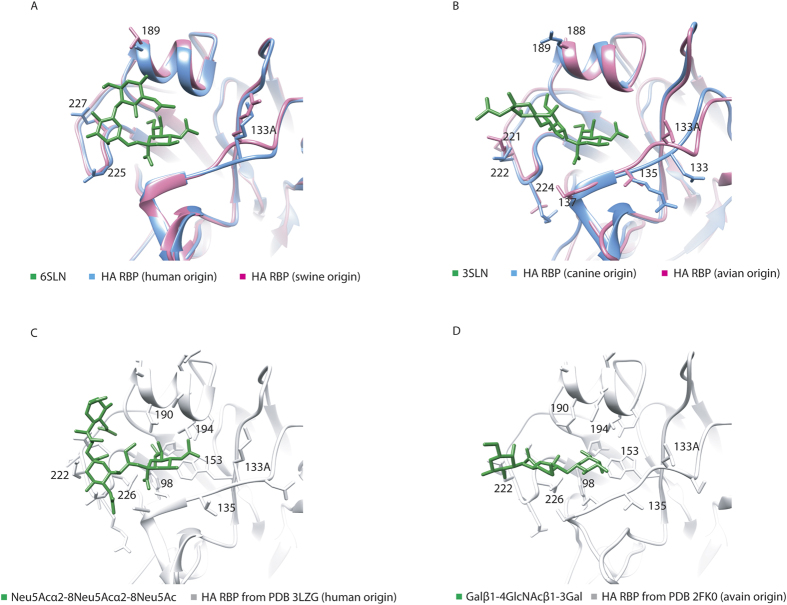
Three-dimensional structures of different hemagglutinin (HA) receptor binding pockets interacting with various glycan substructures. (**A**) Human-origin (Protein Data Bank [PDB] entry 3LZG) and swine-origin (PDB 1RVT) HA (recognizing α2,6 sialic acid) superposed and bound to 6SLN (analogous to Neu5Aca2–6Galb1–4GlcNAc). (**B**) Canine-origin (PDB 4UO5) and avian-origin (PDB 2FK0) HA (recognizing α2,3 sialic acid) superposed and bound to 3SLN (analogous to Neu5Aca2–3Galb1–4GlcNAc). (**C**) A predicted docked structure of Neu5Acα2–8 Neu5Acα2–8Neu5Ac interacting with HA receptor binding pocket (human-origin, PDB 3LZG). (**D**) A predicted docked structure of Galβ1–4GlcNAcβ1–3Gal interacting with HA receptor binding pocket (avian-origin, PDB 2FK0).

In Fig. 6A,B, we show superposed HA receptor binding pockets of different influenza viruses (human-origin with swine-origin viruses and canine-origin with avian-origin viruses) interacting with 6SLN and 3SLN (analogous to glycan substructures). Human- and swine-origin HAs recognize glycans with α2,6-linked sialic acid terminals, and they share a very conserved receptor binding pocket, which differs by only four amino acid residues (133A, 225, 227, and 189) for the different host type viruses (Fig. 6A). Similarly, canine- and avian-origin HAs recognize glycans with α2,3-linked sialic acid terminals, and they also have a conserved receptor binding pocket, but with more diverse residues (133A is deleted on canine HA, and residues differ at amino acids 135, 137, 221, 222, 224, 188, and 189) (Fig. 6B).

In Fig. 6C,D, we docked Neu5Acα2–8Neu5Ac and Galβ1–4GlcNAcβ1–3Gal to the receptor binding pocket of the human-origin HA (PDB 3LZG) and the avian-origin HA (PDB 2FK0) separately by using a HA-glycan structural complex as the template (see Methods). Previous association results suggested a relationship of Neu5Acα2–8Neu5Ac and Galβ1–4GlcNAcβ1–3Gal with the binding for influenza A viruses; thus, their comparable binding poses are expected to occur at the virus HA binding pockets (Fig. 6C,D).

## Discussion

The objective of this study was to characterize the host-specific glycan substructure responding to influenza A virus infections. Glycan microarray data provide an opportunity to systematically study the factors that determine virus–glycan binding. However, such analyses have several limitations. The first limitation is that glycan microarray data are not quantitative because values from batch to batch are highly variable. The variability is caused by spot intensities dependent on immobilization efficiency and results in the misleading use of fluorescence intensities to quantify binding affinities^51^. The second limitation is that the glycans on microarray do not represent all glycans or all substructures in the natural hosts, and they are also distributed differently from those in nature. The last limitation is that the number of datasets for influenza A viruses from viruses of different host origins are not equal. For example, we have 155 datasets for human-origin influenza A viruses but only 7 for canine-origin influenza A viruses.

In this study, we expected association analysis to detect significantly nonrandom, but possibly infrequent, substructure features contributing to influenza A virus binding. To ensure better coverage of all potential substructures, hierarchical clusters (mono-, di-, tri-, and tetra-) of substructure profiles were characterized and integrated into data mining, and our analyses focused on the terminal structures. To minimize the potential noise across different datasets due to variations in glycan microarray versions and experiments, we integrated the significant substructures extracted from each individual dataset by PLS regression. To identify the host-associated glycan substructures, we categorized 211 data entries into five categories (human, swine, canine, waterfowl, and terrestrial birds) and then formulated glycan substructure problems as a typical association mining problem, where we treated glycan substructure features as products, virus host types as the only label of customs, and the glycan–virus binding signals in the dataset as transactions. Comparing to other methods, either statistical or mining strategies, our formulation of the problem benefits the novel observations in this study in two following ways. First, after the PLS regressions on individual glycan microarray entries, the binding transection definition was used to integrate all of them for a cross-array analysis, by which we overcame the challenges from the varying numbers of glycans on different version of arrays. Second, the association mining strategy avoided particular hypothesis before analyses and were able to detect rare but potentially significant rules.

We have not been able to use this method to identify the specific substructures for glycan bindings when multiple terminal glycans are present. For example, glycans with different terminals (e.g. sialic acid and Gal) were observed frequently, but they may both be important players during influenza virus binding because they could bind influenza viruses simultaneously. To avoid this problem, in this study, we ignored the associated substructures with branch linkages, because they may be extracted from a glycan with other terminals and by themselves may not contribute to virus binding. To avoid such false-positives, we included in the results only terminal substructures without branches. Moreover, four substructure definitions (mono-, di-, tri-, tetrasaccharide) could lead to overlapped glycan features that were associated with the same virus host. For example, in Fig. 2, swine-associated disaccharides are all subsets of the corresponding trisaccharides, which are subsets of corresponding tetrasaccharides. Similar patterns could be observed with other host-origin categories (Table S9). To be consistent, we interpreted these overlapped rules by ignoring subset features and by keeping only substructures with the highest number of saccharide residues (see Supplementary Methods).

Our results show that (1) human-origin influenza A viruses could bind glycans with Neu5Acα2–8Neu5Acα2–8Neu5Ac and Neu5Gcα2–6Galβ1–4GlcNAc substructures; (2) Galβ and GlcNAcβ terminal substructures, without any existing sialic acid terminals, are associated with the glycan binding of human-, swine-, and avian-origin influenza A viruses; (3) Sulfated Neu5Acα2–3 substructures are believed to be associated with the glycan binding of human- and swine-origin influenza A viruses. These observations, on one hand, are consistent with previously reported results about various types of host-origin influenza A viruses^5^. On the other hand, we also identified other substructures: α2,6-linked Neu5Gc substructures, α2,8-linked multiple sialic acids, substructures with a Gal and GlcNAc terminals, and sulfated α2,3-linked Neu5Ac, which contribute to different virus bindings. These newly discovered influenza A binding moieties, particularly those with the non-sialic acidic saccharides (Gal, GlcNAc), may suggest that it is the structural pattern of acidic acids, instead of just Neu5Ac, Neu5Gc themselves, which are recognized by influenza viruses of various host origins.

The potential glycan receptors with α2,8-linked sialic acid were reported to be associated with influenza virus binding^22^, which supports our results with Neu5Acα2–8Neu5Acα2–8Neu5Ac for human influenza viruses. The relatively low 3D structural similarities between this substructures and human-like α2,6-linked sialic acid substructures (Table 4) could imply a potentially novel binding mode for Neu5Acα2–8Neu5Acα2–8Neu5Ac (Fig. 6C). Similarly, it has been reported that glycans with Gal terminals could play a role in some virus receptor binding^52^,^53^. Our association results detailed this conclusion, especially for Galβ1–4GlcNAcβ1–3Gal substructure, by supplying similar structural characteristics to substructures with sialic acid. Concerning the associations detected for sulfated α2,3-linked Neu5Ac, it was reported that sulfated glycan motifs might increase influenza virus binding^34^. Our results further suggest that this sulfation process may lead to a SA2,3Gal binding for a SA2,6Gal-binding virus. It is worth mentioning that all these substructure motifs were infrequent substructures in association rules (Table S9), indicating effectiveness of association mining in this study.

Our three-dimensional structure analysis of representative host-specific substructures showed that for trisaccharides, the shape angle formed by mass centers of three residues could be the key feature that distinguishes α2,6-linked, α2,8-linked and α2,3-linked glycans and their virus host tropisms (Fig. 4A–D). Although recent studies argued that the different torsion angles of residue linkages could be the reason for their diverse chain shapes^29^,^44^, our torsion angle values calculated from the three-dimensional structures did not support a role for torsion angle in forming the overall trisaccharide chain shapes. Hence, we argue that significant host-specific patterns related to glycan shape may become evident if shape angles are measured instead of flexible torsion angles. In addition, for trisaccharides without sialic acid terminals (e.g. Galβ1–4GlcNAcβ1–3Gal), neither torsion angles nor RMSD values could suggest any host-specific patterns from our results. However, since we only found a few unique such glycans associated with influenza viruses, we considered them only as individual cases of virus binding without an identifiable structural feature for host tropism.

## Additional Information

**How to cite this article**: Zhao, N. *et al.* Association analyses of large-scale glycan microarray data reveal novel host-specific substructures in influenza A virus binding glycans. *Sci. Rep.* **5**, 15778; doi: 10.1038/srep15778 (2015).

## Supplementary Material

Supplementary Information

Supplementary Table S1

Supplementary Table S2

Supplementary Table S3

Supplementary Table S4

Supplementary Table S5

Supplementary Table S6

Supplementary Table S7

Supplementary Table S8

Supplementary Table S9

## Figures and Tables

**Table 1:** Glycan-binding microarray data collected for 211 wild-type influenza A virus–specific glycan microarray datasets with complete binding affinity values.

Arrayversions	No. Influenzavirus arrays	Host species				
		Human	Swine	Canine	Avian	
					Waterfowl	Terrestrial
V1.0	7	5	0	0	2	0
V2.0	11	10	0	0	1	0
V2.1	4	4	0	0	0	0
V3.0	10	10	0	0	0	0
V3.1	21	2	1	0	8	10
V3.2	5	4	0	0	0	1
V4.0	33	31	2	0	0	0
V4.1	28	15	13	0	0	0
V4.2	20	9	3	0	6	2
V5.0	68	64	0	4	0	0
V5.1	4	0	2	2	0	0
Total	211	154	21	6	17	13

**Table 2:** Distribution of saccharide residues in the glycan substructures from all glycans on the microarrays.

Residue	No. (%) glycan substructures			
	MonosaccharideN = 249	DisaccharideN = 738	TrisaccharideN = 1,198	TetrasaccharideN = 1,477
Neu5Ac	12 (4.82)	45 (6.10)	92 (7.68)	162 (10.9)
Neu5Gc	4 (1.61)	10 (1.36)	16 (1.34)	18 (1.22)
Gal	44 (17.7)	333 (45.1)	811 (67.7)	1,064 (72.0)
GalNAc	35 (14.1)	166 (22.5)	329 (27.5)	400 (27.1)
Glc	31 (12.4)	109 (14.8)	175 (14.6)	201 (13.6)
GlcNAc	45 (18.1)	364 (49.3)	816 (68.1)	1,206 (81.7)
Fuc	6 (2.41)	43 (5.83)	167 (13.9)	310 (20.9)
Man	28 (11.2)	89 (12.1)	235 (19.6)	558 (37.8)

Abbreviations: Fuc, fucose; Gal, galactose; GalNAc, *N*-acetylgalactosamine; Glc, glucose; GlcNAc, *N*-acetylglucosamine; Man, mannose; Neu5Ac, *N*-acetylneuraminic acid; Neu5Gc, *N*-glycolylneuraminic acid.

**Table 3:** Distribution of saccharide residues in the glycan substructures selected by using the quantitative structure–activity relationship.

Residue	No. (%) glycan substructures			
	MonosaccharideN = 73 (out of 249)	DisaccharideN = 230 (out of 738)	TrisaccharideN = 322 (out of 1,198)	TetrasaccharideN = 320 (out of 1,477)
Neu5Ac	7 (9.59)	26 (11.3)	58 (18.0)	102 (31.9)
Neu5Gc	1 (1.37)	3 (1.30)	5 (1.55)	6 (1.88)
Gal	13 (17.8)	107 (46.5)	241 (74.8)	264 (82.5)
GalNAc	10 (13.7)	54 (23.5)	84 (26.1)	76 (23.8)
Glc	4 (5.48)	13 (5.65)	26 (8.07)	32 (10.0)
GlcNAc	15 (20.5)	146 (63.5)	242 (75.2)	268 (83.8)
Fuc	0 (0.00)	7 (3.04)	24 (7.45)	41 (12.8)
Man	12 (16.4)	27 (11.7)	38 (11.8)	79 (24.7)

Abbreviations: Fuc, fucose; Gal, galactose; GalNAc, *N*-acetylgalactosamine; Glc, glucose; GlcNAc, *N*-acetylglucosamine; Man, mannose; Neu5Ac, *N*-acetylneuraminic acid; Neu5Gc, *N*-glycolylneuraminic acid.

**Table 4:** All-against-all RMSD values of representative influenza A host-specific glycan trisaccharide substructures.

Glycan substructure names		RMSD 2value, Å	RMSD 3value, Å
Substructure 1	Substructure 2		
Neu5Acα2-6Galβ1-4GlcNAc	Neu5Acα2-8 Neu5Acα2-8Neu5Ac	3.13	5.58
Neu5Acα2-6Galβ1-4GlcNAc	Neu5Acα2-3Galβ1-4GlcNAc	3.32	7.15
Neu5Acα2-6Galβ1-4GlcNAc	Neu5Gcα2-3Galβ1-4GlcNAc	2.86	7.26
Neu5Acα2-6Galβ1-4GlcNAc	Galβ1-4GlcNAcβ1-3Gal	2.78	8.97
Neu5Acα2-8 Neu5Acα2-8Neu5Ac	Neu5Acα2-3Galβ1-4GlcNAc	5.08	8.90
Neu5Acα2-8 Neu5Acα2-8Neu5Ac	Neu5Gcα2-3Galβ1-4GlcNAc	3.15	4.86
Neu5Acα2-8 Neu5Acα2-8Neu5Ac	Galβ1-4GlcNAcβ1-3Gal	3.19	7.83
Neu5Acα2-3Galβ1-4GlcNAc	Neu5Gcα2-3Galβ1-4GlcNAc	3.29	8.01
Neu5Acα2-3Galβ1-4GlcNAc	Galβ1-4GlcNAcβ1-3Gal	2.99	6.44
Neu5Gcα2-3Galβ1-4GlcNAc	Galβ1-4GlcNAcβ1-3Gal	1.94	4.38

Abbreviations: RMSD, root-mean-square deviation; RMSD2, the RMSD between the two six-membered rings of the saccharides linked to the terminal saccharide; RMSD3, the RMSD between them of the third pair of saccharides.

**Table 5:** Torsion angles (ϕ and ψ) of linkage 1 and linkage 2 of representative influenza A host-specific glycan substructures.

Glycan substructure name	Linkage 1		Linkage 2	
	ϕ	ψ	ϕ	ψ
Neu5Acα2-6Galβ1-4GlcNAc	71.32	−151.25	−94.84	82.75
Neu5Acα2–8 Neu5Acα2–8Neu5Ac	55.05	112.07	57.35	121.40
Neu5Acα2–3Galβ1–4GlcNAc	−59.47	−126.54	−81.61	124.23
Neu5Gcα2–3Galβ1–4GlcNAc	50.95	−134.56	−67.73	105.46
Galβ1–4GlcNAcβ1–3Gal	−96.61	95.29	−46.87	−139.85
