# Supplementary material for: Association analyses of large-scale glycan microarray data reveal novel host-specific substructures in influenza A virus binding glycans
Source: Sci Rep. 2015 Oct 28;5:15778. doi: 10.1038/srep15778 (PMC4623813; doi:10.1038/srep15778)
Supplement: Supplementary Information [file srep15778-s1.pdf]

Supplementary Information

Association analyses of large-scale glycan microarray data reveal novel host-specific substructures in influenza A virus binding glycans

Nan Zhao[1,2], Brigitte E. Martin[1], Chun-Kai Yang[1], Feng Luo[3], Xiu-Feng Wan[1,2] *

[1] Department of Basic Sciences, College of Veterinary Medicine, Mississippi State University, MS, USA
[2] Institute for Genomics, Biocomputing & Biotechnology, Mississippi State University, MS, USA
[3] School of Computing, Clemson University, Clemson, SC, USA
*Address correspondence to wan@cvm.msstate.edu

Supplementary methods

Glycan substructure feature extraction

Glycan substructures were defined as described elsewhere[1]. Specifically, mono-, di-, tri-, and tetrasaccharide substructures were extracted from 936 glycans as features. These extractions resulted in 249 monosaccharide, 738 disaccharide, 1,198 trisaccharide, and 1,477 tetrasaccharide substructures (S4-S7 Tables). The fluorescent signal value for the corresponding glycan on the array was assigned as the binding affinity for each individual substructure. Only fluorescent signal values ≥ 2,000 were considered as effective numbers in regression, and those <2,000 were treated as background noise.

A partial least squares (PLS) regression and feature selection algorithm[1] were adapted to select the features predominating glycan binding from an influenza virus–specific glycan microarray dataset. In brief, by using binding affinities as responses and substructure features as predictors, PLS regression was used to establish the relationship between them. In mathematical terms, if vector y was the binding affinity response and matrix X was the substructure features, latent variables were first denoted by a matrix T, which was a linear combination of X: $T_{ij} = \sum_k W_{kj} X_{ik}$. After that, y responses were expressed by T: $y_m = \sum_j C_{mj} T_{ij} + f_m$, where matrix C was the coefficients of T for y and vector f held random errors between X and y.

For each single PLS regression on substructure features and binding affinity responses, a significant feature selection was done by using the PLS-β method, which calculated the coefficient matrix B of the regression model $y_m = \sum_k B_{mk} X_{ik} + f_m^{'}$ by $B_m = \sum_i C_{mi} W_{ki}$ [2]. This PLS regression was performed four times for each single data entry from our 211 glycan microarrays by using four sets of substructure feature definitions. In this way, a single data entry from 211

glycan microarrays resulted in four sets of significant glycan substructures: mono-, di-, tri-, and tetrasaccharides. Each feature vector was labeled according to the host origin of the influenza A virus used in the glycan microarray experiments (i.e., human, swine, canine, waterfowl, or terrestrial bird [chicken, quail, and turkey] host).

Association rule mining for selected glycan substructures

Association rule mining (learning) has been widely used in the field of marketing analysis for discovering regularities between products from a large-scale database of transaction records[3-6]. Unlike hypothesis testing, which uses statistical methods such as ANOVA and which has been widely used to interpret glycan microarray data[7-11], association analysis does not require particular hypotheses. Instead, association analysis mines all relationships from a large number of features at the same time at a given confidence level. We formulated the detection of host-specific glycan substructures as an association-mining task that discovers rules between substructure features and virus host types.

As an example of this rules-discovering association-mining task, let items $I = \{i_1, i_2, \cdots, i_n\}$ represent a set of items and let $T = \{t_1, t_2, \cdots, t_m\}$ be a set of transactions forming a database. Each transaction in T has a subset of items in I. An association rule, $X \Rightarrow Y$, where $X, Y \subseteq I$, is usually interpreted to mean that when the items in X exist, those in Y also occur at a certain confidence level[12]. Here, for our glycan microarray dataset, transactions *T* were the data derived from influenza virus–specific glycan microarray entries, so m=211; the substructure features *X* derived from glycans on the array by previous PLS-β selection and the labeled features *Y* with host origin will form *I*. We aimed to detect association rules *X* that determine *Y*.

Given a rule $X \Rightarrow Y$, the *confidence* is defined as $Conf(X \Rightarrow Y) = supp(X \cup Y) / supp(X)$, where $supp(X)$ is the *support* of item set X. The *support* was defined as the proportion of transactions in the dataset, which contains the item set. Another measurement, *lift*, is the ratio of the observed *support* and was defined as $Lift(X \Rightarrow Y) = supp(X \cup Y) / (supp(X) \times supp(Y))$[12]. Therefore, we expected to obtain interesting association rules with high confidences (≥ 80%), high lifts (has a lift value ≥ 1[13]), and low supports (infrequent but potentially interesting) to supply highly probable, unexpected, and infrequent conclusions. We adapted the *Apriori* algorithm implemented in R[14] to infer these host substructure–specific associations and the corresponding confidence level. The Apriori algorithm is a classic association mining strategy that discovers rules from a given transaction dataset through, first, finding frequent item sets by the minimum support value, and then, forming rules by limiting minimum confidence on these frequent item sets. In this study, the support threshold was set to a low value of 0.005 to detect as many qualifying rules as possible, and the confidence threshold was set at 80%. Moreover, during the mining process, redundant rules were also removed by defining super rules as redundancy. A super rule is a rule with the same or lower lift value, where the left hand side, *X*, contains more items than a previous rule, but still results in the same right hand side, Y. We kept only satisfied rules, which were filtered by leaving only those with terminal saccharides on the substructure features. Because we extracted mono-, di-, tri-, and tetrasaccharide substructures and they were mined as independent features, the resulting rules could have overlapping substructures on the left hand side. For example, Neu5Acα2-6Galβ1, is overlapped with

Neu5Acα2-6Galβ1-4GlcNAcβ. To keep the most specific glycan substructure feature patterns, for all subsequent associations, we only reserved the maximum level glycan substructure containing all substructures identified in lower-level substructure mining. That is, for example, if both monosaccharide and disaccharide substructures are predicted as significant and if monosaccharide substructure is a part of disaccharide substructure, only the disaccharide substructure was defined as the effective rule. In the preceding example, we would use Neu5Acα2-6Galβ1-4GlcNAcβ instead of Neu5Acα2-6Galβ1 if both were significant in association rule mining.

Supplementary References

1 Xuan, P., Zhang, Y., Tzeng, T. R., Wan, X. F. & Luo, F. A quantitative structure-activity relationship (QSAR) study on glycan array data to determine the specificities of glycan-binding proteins. *Glycobiology* **22**, 552-560, doi:10.1093/glycob/cwr163 (2012).

2 Chong, I.-G. & Jun, C.-H. Performance of some variable selection methods when multicollinearity is present. *Chemometrics and Intelligent Laboratory Systems* **78**, 103-112 (2005).

3 Jiao, J. & Zhang, Y. Product portfolio identification based on association rule mining. *Computer-Aided Design* **37**, 149-172 (2005).

4 Wong, K. W., Zhou, S., Yang, Q. & Yeung, J. M. S. Mining customer value: From association rules to direct marketing. *Data Mining and Knowledge Discovery* **11**, 57-79 (2005).

5 Chen, M.-C., Chiu, A.-L. & Chang, H.-H. Mining changes in customer behavior in retail marketing. *Expert Systems with Applications* **28**, 773-781 (2005).

6 Joshi, A. & Sodhi, J. Target Advertising via Association Rule Mining. *International Journal* **2** (2014).

7 Kiwamoto, T. *et al.* Mice deficient in the St3gal3 gene product alpha2,3 sialyltransferase (ST3Gal-III) exhibit enhanced allergic eosinophilic airway inflammation. *The Journal of allergy and clinical immunology* **133**, 240-247 e241-243, doi:10.1016/j.jaci.2013.05.018 (2014).

8 Yen, H. L. *et al.* Hemagglutinin-neuraminidase balance confers respiratory-droplet transmissibility of the pandemic H1N1 influenza virus in ferrets. *Proceedings of the National Academy of Sciences of the United States of America* **108**, 14264-14269, doi:10.1073/pnas.1111000108 (2011).

9 Watanabe, T. *et al.* Characterization in vitro and in vivo of pandemic (H1N1) 2009 influenza viruses isolated from patients. *Journal of virology* **86**, 9361-9368, doi:10.1128/JVI.01214-12 (2012).

10 Bleuler-Martinez, S. *et al.* A lectin-mediated resistance of higher fungi against predators and parasites. *Molecular ecology* **20**, 3056-3070, doi:10.1111/j.1365-294X.2011.05093.x (2011).

11 Sun, X. *et al.* N-linked glycosylation of the hemagglutinin protein influences virulence and antigenicity of the 1918 pandemic and seasonal H1N1 influenza A viruses. *Journal of virology* **87**, 8756-8766, doi:10.1128/JVI.00593-13 (2013).

12 Hand, D. J., Mannila, H. & Smyth, P. *Principles of data mining*. (MIT press, 2001).

13 Geng, L. & Hamilton, H. J. Interestingness measures for data mining: A survey. *ACM Computing Surveys (CSUR)* **38**, 9 (2006).

14 Borgelt, C. & Kruse, R. in *Compstat.* 395-400 (Springer).

Supplementary table titles

Table S1. Glycan microarray data entries of 211 wild-type influenza viruses.

Table S2. Merged list of 936 unique glycans from 11 versions of microarrays.

Table S3. Original binding affinities from glycan microarrays of 211 data entries.

Table S4. Glycan substructure feature names of monosaccharides.

Table S5. Glycan substructure feature names of disaccharides.

Table S6. Glycan substructure feature names of trisaccharides.

Table S7. Glycan substructure feature names of tetrasaccharides.

Table S8. Significant glycan substructures selected by QSAR (quantitative structure–activity relationship).

Table S9. Association rules grouped by host type and ranked by lift and frequency values. Terminal glycan substructures are shown on the left, and virus host types are shown on the right.
